# The Inner Ear Heat Shock Transcriptional Signature Identifies Compounds That Protect Against Aminoglycoside Ototoxicity

**DOI:** 10.3389/fncel.2018.00445

**Published:** 2018-11-23

**Authors:** Matthew Ryals, Robert J. Morell, Daniel Martin, Erich T. Boger, Patricia Wu, David W. Raible, Lisa L. Cunningham

**Affiliations:** ^1^Section on Sensory Cell Biology, National Institute on Deafness and Other Communication Disorders, National Institutes of Health, Bethesda, MD, United States; ^2^Department of Molecular Biology and Genetics, Johns Hopkins University School of Medicine, Baltimore, MD, United States; ^3^Genomics and Computational Biology Core, National Institute on Deafness and Other Communication Disorders, National Institutes of Health, Bethesda, MD, United States; ^4^Oral and Pharyngeal Cancer Branch, National Institute of Dental and Craniofacial Research, National Institutes of Health, Bethesda, MD, United States; ^5^Virginia Merrill Bloedel Hearing Research Center, University of Washington, Seattle, Seattle, WA, United States; ^6^Department of Biological Structure, University of Washington, Seattle, Seattle, WA, United States

**Keywords:** library of integrated cellular signatures (LINCS), drug screening and discovery, hearing loss, ototoxicity, RNA-Seq

## Abstract

Mechanosensory hair cells of the inner ear transduce auditory and vestibular sensory input. Hair cells are susceptible to death from a variety of stressors, including treatment with therapeutic drugs that have ototoxic side effects. There is a need for co-therapies to mitigate drug-induced ototoxicity, and we showed previously that induction of heat shock proteins (HSPs) protects against hair cell death and hearing loss caused by aminoglycoside antibiotics in mouse. Here, we utilized the library of integrated cellular signatures (LINCS) to identify perturbagens that induce transcriptional profiles similar to that of heat shock. Massively parallel sequencing of RNA (RNA-Seq) of heat shocked and control mouse utricles provided a heat shock gene expression signature that was used in conjunction with LINCS to identify candidate perturbagens, several of which were known to protect the inner ear. Our data indicate that LINCS is a useful tool to screen for compounds that generate specific gene expression signatures in the inner ear. Forty-two LINCS-identified perturbagens were tested for otoprotection in zebrafish, and three of these were protective. These compounds also induced the heat shock gene expression signature in mouse utricles, and one compound protected against aminoglycoside-induced hair cell death in whole organ cultures of utricles from adult mice.

## Introduction

Hair cells are the sensory receptors of the inner ear and are susceptible to damage by a variety of stressors, referred to as ototoxins. A few lifesaving therapeutic drugs have ototoxic properties. The aminoglycoside antibiotics such as gentamicin and neomycin, used to treat drug-resistant tuberculosis and other severe infections, are one major class of widely used therapeutics with ototoxic side effects. Other widely used ototoxic drugs include the platinum-containing antineoplastic agents such as cisplatin, which is used to treat solid tumors. The hearing and vestibular damage associated with these drugs ranges from approximately 20–30% in patients receiving aminoglycoside antibiotics (Moore et al., 1984; Lerner et al., 1986; Fausti et al., 1999) to as high as 75–100% of patients receiving the chemotherapeutic cisplatin (McKeage, 1995).

The inner ear is capable of generating intrinsic protective signaling mechanisms to prevent the death of hair cells. Induction of heat shock proteins (HSPs) using a heat shock stress can prevent both aminoglycoside- and cisplatin-induced ototoxicity in mouse utricles *in vitro* (Cunningham and Brandon, 2006), and this protection is dependent on the heat-inducible form of heat shock protein 70 (HSP70i) (Taleb et al., 2008). The heat shock response can also be induced by the pharmacological inhibition of HSP90, which induces the response by releasing the transcription factor heat shock factor 1, (HSF1; Whitesell et al., 2003). Protection against ototoxicity, referred to as otoprotection, using HSP90 inhibitors has been reported in *in vitro* experiments of rat inner ear tissue exposed to gentamicin and treated with HSP90 inhibitor geldanamycin (Yu et al., 2009) and in mouse inner ear tissue exposed to kanamycin treated with HSP90 inhibitor alvespimycin (Liu Y. et al., 2015). Pharmacological induction of other HSPs also render otoprotection *in vivo* as was previously shown in guinea pigs exposed to systemic cisplatin given geranylgeranylacetone, which induced three different families of HSPs (HSP27, HSP40, and HSP70) and reduced cisplatin-induced hearing loss (Lo et al., 2017). Some closely related stress-induced proteins, such as heme oxygenase I (HO-1 aka HSP32) are also otoprotective *in vitro* and *in vivo* (Francis et al., 2011; Baker et al., 2015). Thus, there is ample evidence demonstrating that HSP induction is otoprotective, and the identification of compounds that mimic heat shock has the potential to advance the development of therapies to prevent hearing loss associated with ototoxic drugs.

While there is no cell line that appropriately represents sensory hair cells, the zebrafish (*Danio rerio*) lateral line has proven a useful model system for screening compounds for their effects on sensory hair cells. Zebrafish have hair cells grouped into neuromasts, which the animal uses to detect changes in water current (Ou et al., 2010). Ototoxic drug-induced hair cell death in the zebrafish neuromast is well-characterized in response to a variety of ototoxic compounds including cisplatin, gentamicin, and neomycin (Harris et al., 2003; Ton and Parng, 2005). Molecules that are protective against cisplatin-induced ototoxicity in mammals, such as *N*-acetylcysteine and D-methionine, are also protective against cisplatin-induced hair cell death in zebrafish neuromasts, indicating that the zebrafish neuromast is a reasonable model system in which to screen for otoprotective compounds (Ton and Parng, 2005), and substantial medium-throughput screens have been reported. A screen of 1,040 FDA-approved compounds and bioactives for protection against aminoglycoside ototoxicity revealed seven compounds, four of which inhibited hair cell death in zebrafish neuromasts through blocking of aminoglycoside uptake into hair cells and the remaining three through inhibition of hair cell death through cell signaling mechanisms. One of the three compounds that prevent hair cell death was further validated as protective against neomycin in a model system in mouse utricle explant cultures (Ou et al., 2009). Another screen of 640 FDA-approved compounds against neomycin, kanamycin, gentamicin, and cisplatin revealed 10 compounds that were protective against at least two of these ototoxins (Vlasits et al., 2012). Lastly, a screen of 160 ion channel modulators in zebrafish revealed 72 compounds that affected hair cell function, 13 of which protected against gentamicin damage in mouse neonatal cochlear cultures (Kenyon et al., 2017).

The results of another zebrafish neuromast screen identified a novel candidate compound protective against aminoglycoside-induced hair cell death (Owens et al., 2008) that was subsequently modified into an otoprotective compound known as ORC-13661 that is protective in both rats and zebrafish *in vivo* (Chowdhury et al., 2018). Despite the progress made using the zebrafish model, it would be useful to be able to screen compounds in a mammalian cell line. Although a cell line, HEI-OC1, with inner ear cell type-like properties (Kalinec et al., 2003) has been developed, these cells have limitations in their cellular death responses and sensitivity to aminoglycoside ototoxicity that limit their use in otoprotection studies (Cederroth, 2012; Chen et al., 2012; Kalinec et al., 2016). There are other cell models currently being developed for use in high-throughput drug screening (Kwan et al., 2015; Walters et al., 2015). The otoprotective effect of inducing HSPs through both physiological and pharmacological means suggests that these treatments share a transcriptional pattern of HSP gene expression. Knowledge of a shared transcriptional pattern among these treatments may help identify a protected cellular state capable of preventing hair cell death.

The connectivity map (CMAP) project was developed with the goal of identifying transcriptional patterns among 164 small molecule treatments in three cell lines using gene expression microarrays (Lamb et al., 2006). The results of CMAP were made publicly available, and this allowed investigators to query whether CMAP-tested small molecules induce similar or reverse gene expression patterns compared to disease states they might be investigating. Thus, an investigator would either be able to find compounds that could produce similar beneficial expression profiles to their biological transcriptional state of interest, or compounds that could oppose or even reverse transcriptional expression patterns associated with certain active disease expression profiles. Queries that aligned disease state to expression pattern using CMAP led to several advances, including the re-purposing of the anthelmintic microtubule polymerization inhibitor parbendazole as a potential osteoporosis therapeutic and the use of celastrol as a leptin sensitizer to treat obesity in mice (Brum et al., 2015; Liu J. et al., 2015). The original CMAP project was expanded using the L1000 gene expression assay (Peck et al., 2006) to increase the number of compounds and cell numbers screened as part of the NIH Library of Integrated Network-based Cellular Signatures (LINCS) initiative. In its most recent iteration, LINCS has 19,811 small molecule profiles and 5,075 gene knockdown/overexpression profiles assayed in 77 cell lines (Subramanian et al., 2017). Investigators generating the LINCS profiles have also expanded the LINCS query tool to include a subset of this expanded dataset. As with the CMAP data, the query tool can show an investigator if there are any LINCS L1000-profiled small molecules that produce similar or opposite effects to the input gene expression pattern of interest. In our study, we utilized the LINCS gene expression query tool to generate a list of perturbagens that matched the heat shock response gene signature in the inner ear.

## Materials and Methods

### Animals

Male and female CBA/J mice were obtained from The Jackson Laboratory. Young adult mice (age 4–8 weeks) were euthanized by CO_2_ inhalation followed by decapitation. Mouse animal protocols were approved by the NIDCD Institutional Animal Care and Use Committee. Five to seven days post fertilization (dpf) zebrafish larvae (wildtype, ^∗^AB strain) were maintained at 28.5°C. Starting at 5 dpf, fish were anesthetized using MS222 (tricaine methanesulfonate, Sigma) and imaged either live or after fixation for 2 h with 4% paraformaldehyde. Zebrafish procedures were approved by the University of Washington Animal Care and Use Committee.

### Organotypic Utricle Explant Culture

Utricles were dissected from both male and female CBA/J mice (age 4–8 weeks) into sterile 24-well plates as free-floating cultures (five to six utricles from two to three animals pooled per well). Utricles were cultured in an incubator overnight in culture medium [DMEM/F12 media supplemented with 5% fetal bovine serum (FBS), Life Technologies] and 50 U/ml penicillin G) at 37°C (95% air/5% CO_2_). For induction of the heat shock response, utricles and medium were transferred to a sterile 1.5 mL centrifuge tube that was placed in a water bath at 43°C for 30 min. Utricles were then returned to the 24-well plate and recovered under culture conditions (37°C) for 2 h for heat shock mRNA induction before downstream processing to extract RNA. For LINCS perturbagen gene expression tests, utricles were incubated overnight in culture medium at 37°C (95% air/5% CO_2_), followed by transfer into solutions containing perturbagens or a vehicle (0.1% DMSO). Following a 6-h incubation in each perturbagen, utricles were immediately processed for RNA extraction. For LINCS perturbagen neomycin protection assays, utricles were incubated overnight in culture medium at 37°C (95% air/5% CO_2_), and were then exposed to each perturbagen for 6 h followed by a brief 5-min washout in culture medium. They were then exposed to 2.5 mM neomycin for 24 h. Neomycin was prepared in culture medium and equilibrated at 37°C and 5% CO_2_ for 3–6 h before utricles were transferred. Following neomycin exposure, utricles were fixed and processed for immunohistochemistry.

### RNA Extraction and Quality

RNA was extracted from four to six utricles according to the RNaqueous Micro kit protocol (Ambion). DNase I enzyme treatment was performed on each extracted RNA sample using the protocol in the RNaqueous DNase I kit to remove residual genomic DNA. All RNA samples were then analyzed using a Bioanalyzer (Agilent) and a total RNA Pico Chip (Agilent) to assess RNA integrity number (RIN) score and RNA concentration, and for subsequent normalization of concentration for reverse transcription and qPCR. RNA samples with RIN scores of ≥8 were used in subsequent qPCR assays. There were some exceptions to this criterion in the RNA-Seq validation group (*n* = 3 per treatment, four to six utricles per well, two to three mice total used per treatment replicate), where the third control replicate used had a RIN score of 5.5 but did not show significantly different *C*t values compared to the other control replicates. The exception to the RIN criterion in the perturbagen qPCR experiments was the third biological replicate of AT13387 exposure in utricles, which had a RIN score of 6.3; however, there was no noticeable difference in the fold induction pattern or *C*t values observed from this replicate compared to replicates with higher RIN values, so it was included in the dataset. For RNA-Seq, RIN scores and concentrations were analyzed, and all replicates used had RIN scores of ≥8 (*n* = 4 wells per treatment, four to six utricles per well, two to three mice total per treatment). The fourth control replicate had a RIN score of 4.5 and was dropped from subsequent analyses after RNA-Seq alignment, as it was an outlier library that had poor alignment compared to the other three control replicates.

### cDNA/Library Preparation and RNA-Sequencing

Double-stranded cDNA was prepared using the SMART-Seq v4 Ultra Low Input Kit (Clontech). Libraries were prepared using a Nextera XT (Illumina) kit, individually barcoded, pooled to a 2 nM final pooled concentration, and sequenced on a HiSeq 1500 (Illumina) using 125 × 125 paired-end mode (trimmed to 93 × 93). Reads were aligned to the GENCODE vM4 mouse genome (GRCm38.p3) using STAR (v2.4.2a) (Dobin et al., 2013). Consensus heat shock gene expression signatures were generated by selecting those genes that three different gene expression (DGE) analysis tools identified as being significantly enriched or depleted: The analysis tools used were DESeq2 (Love et al., 2014), EdgeR (Robinson et al., 2010; McCarthy et al., 2012), and Limma-voom (Law et al., 2014). EdgeR and Limma-voom DEG tables were generated using Degust (Powell, 2015).

### LINCS Query Tool

Differential gene expression (DEG) analyses were performed on the heat shocked utricle RNA-Seq data generated lists of transcripts that were either enriched or depleted by heat shock. The lists were then entered into the LINCS query tool on the LINCS Cloud website. At the time of the analysis the query tool was hosted on LINCS Cloud^1^, and as of this publication the query tool is now hosted on the CLUE Platform^2^. The LINCS data used in the query are also available in two GEO repositories (GSE92742 and GSE70138). The query tool identified transcripts from each list that it recognized based on its own directly measured and computationally inferred gene lists as represented by the red and blue lines in the LINCS query flowchart (see Results, Figure 3A). The transcripts that were recognized by the query tool are summarized in Supplementary Table S1.

### RT-qPCR Expression

RNA extracted from utricles was reverse transcribed to cDNA using Taqman Reverse Transcription Reagents (Applied Biosystems), and gene targets were measured using Taqman assays normalized to *Actb* (primer-limited) multiplexed with target Taqman gene assays. qPCRs were performed on a 7500 Real Time PCR system (Applied Biosystems) for perturbagen exposure testing and RNA-Seq DEG validation, with plates prepared with Taqman Gene Expression PCR Master Mix (2X) (Applied Biosystems). Applied Biosystems qPCR results for RNA-Seq validation genes were also run independently on a Biomark HD platform using a Flex Six^TM^ integrated fluidic circuit (IFC) (Fluidigm) according to manufacturer instructions and normalized to *Gapdh* (non-multiplexed) for fold change calculations. Briefly, cDNA samples underwent a 14-cycle PCR preamplification using relevant Taqman primers to amplify target cDNA. The Flex Six^TM^ IFC was then primed with control line fluid using the IFC Controller HX (Fluidigm). Pre-amplified cDNA, 20X Gene Expression Master Mix (Fluidigm), Taqman Gene Expression PCR Master Mix (2X), Taqman gene expression assays, and 2X Assay Loading Reagent were then loaded onto the primed IFC, which was then run on the Biomark HD. All Taqman assays used for qPCR experiments are listed in Table 1. For perturbagen gene expression testing, utricles were exposed to individual doses of perturbagen dissolved in 0.1% DMSO and compared to control utricles treated only with the 0.1% DMSO vehicle. For additional comparison, the profile of a 2-h exposure to 0.1% DMSO vehicle was performed from *C*t values obtained from the Biomark HD from vehicle-treated utricles run on the same IFC partition compared to the no heat-shocked control utricle samples from the RNA-Seq DEG validation experiment. Biological triplicate replicates (*n* = 3 wells, four to six utricles per well, two to three mice per treatment) were performed for vehicle groups and perturbagen-exposed groups. For heat shock RNA-Seq DEG validation, biological triplicate replicates (*n* = 3 wells, four to six utricles per well, two to three mice per treatment) for non-heat shocked and heat shocked groups were performed.

**Table 1:** Summary table of gene names and Taqman assay product identifiers used for validation by RT-qPCR in utricle of the DEG heat shock signature identified by RNA-Seq (Figure 2) and LINCS perturbagen gene expression profiling in utricle (Figure 6).

Gene name	Taqman assay ID
*Hspa1b*	Mm03038954_s1
*Hspa1a*	Mm01159846_s1
*Hspb1*	Mm00834384_g1
*Hsph1*	Mm00442864_m1
*Dnajb1*	Mm 00444519_m1
*Bag3*	Mm00443474_m1
*Cacybp*	Mm01295897_g1
*Chac1*	Mm00509926_m1
*Mgp*	Mm00485009_m1
*Tnfsf10*	Mm01283606_m1
*Gjc3*	Mm01204089_m1
*Hmox1*	Mm00516005_m1
*Hspe1*	Mm00434083_m1
*Actb*	Mm02619580_g1
*Gapdh*	Mm99999915_g1


### Immunohistochemistry and Imaging

#### Mouse Utricles

Utricle hair cell survival was assessed by counting myosin VIIa positive hair cells in utricles (*n* = 4–12 utricles per treatment from two to six mice) fixed with 4% PFA overnight at 4°C, washed in 1X PBS (3X 15 min washes), blocked in immunohistochemistry (IHC) block buffer (1X PBS, 2% bovine serum albumin, 0.8% normal goat serum or normal donkey serum, and 0.4% Triton X-100) at RT for 3 h. Utricles were immunostained using a mouse anti-myosin VIIa primary antibody (1:100, Developmental Studies Hybridoma Bank, 138-1) overnight at 4°C. Utricles were then washed three times each for 15-min in IHC block buffer, followed by incubation in an anti-mouse Alexafluor-488 conjugated secondary antibody in IHC block buffer (1:500, Thermo Fisher) followed by a 10-min incubation using a nuclear counterstain (1:5000 Hoechst 33342, Thermo Fisher) in 1X PBS followed by three 15-min washes in 1X PBS. Utricles were then mounted on glass slides using Fluoromount G (Southern Biotech). Hair cell counts taken from 50 μm × 50 μm boxes located either within the central (within the line of polarity reversal, a region that demarcates opposing orientations of hair cells in the utricle) or the peripheral (outside of the line of polarity reversal) region of the utricle, with four boxes sampled in each region.

#### Zebrafish

Following exposure to neomycin and perturbagen compound dose responses, zebrafish larvae (*n* = 9–11 larvae per treatment group, aged 5–7 dpf) were fixed with 4% PFA (in 1X PBS) for 2 h at RT, followed by three 15-min washes in 1X PBS. Zebrafish larvae were then incubated for a 2-h blocking period at room temperature (1% Triton X-100, 5% NGS in PBS). Larvae were then immunostained with mouse anti-parvalbumin primary antibody (monoclonal 1:400, Millipore MAB1572) in primary block (1% Triton X-100, 1% NGS in 1X PBS) at 4°C overnight. Following three 15-min washes in PBS-T (1X PBS, 1% Triton X-100), larvae were transferred into a solution containing a goat anti-mouse secondary antibody conjugated to Alexafluor-488 (1:500) in secondary block (1% NGS in 1X PBS). Larvae were washed in three 15-min washes with PBS-T followed by three 15-min washes in 1X PBS. Larvae were mounted using Fluoromount G on glass slides, and hair cell counts were performed on the SO1, SO2, O1, and OC1 neuromasts using an Axioplan fluorescent microscope (Zeiss) at 40× magnification as previously described (Raible and Kruse, 2000; Harris et al., 2003).

### DASPEI Live Imaging

Zebrafish larvae were placed into 48-well plates and cultured in 300 μL embryo medium (EM) (1 mM MgSO_4_, 120 μM KH_2_PO_4_, 74 μM Na_2_HPO_4_, 1 mM CaCl_2_, 500 μM KCl, 15 μM NaCl, and 500 μM NaHCO_3_ in dH_2_O) overnight. Following culture, zebrafish were exposed to EM alone, vehicle (0.1–1% v/v DMSO depending on compound solubility or 0.1% v/v ethanol) alone, vehicle plus 200 μM neomycin, or perturbagen (10 μM, 1 μM, or 30 μM testing concentrations depending on the compound) plus neomycin for 1 h. No toxic effect was observed as a result of vehicle exposure compared to EM alone, and no additional toxicity was observed in addition to neomycin. A list of the 42 screened perturbagens, vendor information, and location identification code for each perturbagen on the screening plate are summarized in Table 2. At the screening stages of the project, the compounds were identified by these plate location codes, thus we refer to these codes when we report the results of the screen. Following exposure to each compound, zebrafish were transferred into a six-well plate basket, washed twice with EM, and incubated in a solution of DASPEI (2-[4-(dimethylamino)styryl]-*N*-ethylpyridinium iodide) for 15 min, washed four times in EM, and placed into a solution of MS222 (tricaine methanesulfonate) for 5 min for anesthesia. DASPEI scoring was performed using a MZ FL III fluorescent stereomicroscope (Leica Microsystems) with a DASPEI filter (excitation filter range: 450–490 nM, and barrier filter at 515 nM; Chroma Technologies) on anesthetized animals as previously described (Harris et al., 2003). Briefly, 10 neuromasts per larva labeled with DASPEI were visualized at 5× magnification and evaluated for integrity based on a 0–2 scoring system. A score of 0 indicated an absence of all hair cells in the neuromast, 1 indicated partial loss of hair cells, and 2 indicated an intact neuromast. Scores from all 10 neuromasts were added together to give a composite DASPEI score for an individual zebrafish larva (*n* = 10 larvae per treatment and *n* = 5–10 larvae for control groups). Anesthetized animals were euthanized in an ice bath (4°C or less) following DASPEI score determination.

**Table 2:** Summary table of 42 perturbagens used the zebrafish DASPEI screen.

Perturbagen name	Vendor (Cat #)	Plate location
Sirolimus	SelleckChem S1039	A1
BIIB021	SelleckChem S1175	A2
CYT-997	SelleckChem S2195	A3
Anisomycin	SelleckChem S7409	A4
Withaferin-a	Tocris 2816	A5
BCI-hydrochloride	Sigma B4313	A6
Arachidonyl-trifluoro-methyl ketone	Tocris 1462	A7
Trichostatin-A	SelleckChem S1045	B1
Etoposide	SelleckChem S1225	B2
Parthenolide	SelleckChem S2341	B3
Piperlongumine	SelleckChem S7551	B4
CMPD-1	Tocris 2186	B5
Elesclomol	SelleckChem S1052	C1
Ranolazine	SelleckChem S1425	C2
Ursolic-acid	SelleckChem S2370	C3
SB-225002	SelleckChem S7651	C4
AEG 3482	Tocris 2651	C5
NVP-AUY922	SelleckChem S1069	D1
AZD-6482	SelleckChem S1462	D2
MG-132	SelleckChem S2619	D3
Xanthohumol	SelleckChem S7889	D4
BNTX maleate	Tocris 0899	D5
Tanespimycin	SelleckChem S1141	E1
Disulfiram	SelleckChem S1680	E2
Fostamatinib	SelleckChem S2625	E3
Butein	SelleckChem S8036	E4
Manumycin-A	Sigma 444170-M	E5
Alvespimycin	SelleckChem S1142	F1
Teniposide	SelleckChem S1787	F2
Geldanamycin	SelleckChem S2713	F3
PU-H71	SelleckChem S8039	F4
Phenethyl-isothiocyanate	Sigma 253731	F5
STA-9090	SelleckChem S1159	G1
Menadione	SelleckChem S1949	G2
Pifithrin-μ	SelleckChem S2930	G3
NSC 632839	Tocris 2647	G4
MLN-4924	EMD Millipore 5.05477.0001	G5
AT-13387	SelleckChem S1163	H1
MLN-2238	SelleckChem S2180	H2
PP-1	SelleckChem S7060	H3
SA-792709	Tocris 2020	H4
Sappanone a dimethyl ether	MicroSource 201136	H5


*Table contains the name of each perturbagen compound, the vendor and catalog number of the compound purchased for the screen, and its location on the screening plate used as the stock concentration plate, which was used to determine perturbagen identity after screening.*

### Statistics

Statistical analyses and data visualizations were performed either in R for DGE analysis tools [including Corrplot (Wei et al., 2017) for global correlation visualization, PCAExplorer (Marini, 2018) for DESeq2 PCA, heatmap, and PC gene loading visualizations] or Graphpad Prism 6 (GraphPad Software) for all other analyses. Statistical significance for zebrafish DASPEI scores was determined using the non-parametric Kruskal–Wallis test with Dunn’s multiple comparisons comparing all controls and perturbagen treatments to the neomycin-only treated group. Statistical significance for zebrafish neuromast and mouse utricle hair cell counts were determined using one-way ANOVA followed by Tukey or Sidak multiple comparison *post hoc* tests. Statistical significance for perturbagen qPCR fold changes was performed using multiple unpaired *t*-tests on Δ*C*t values between treatment groups with Holm-Sidak significance correction for multiple comparisons, and relative quantification of fold changes using the ΔΔ*C*t method were then plotted. Correlations between qPCR and RNA-Seq fold changes were performed using a two-tailed Pearson correlation test. Graphs are shown with mean values ± standard deviation values unless otherwise noted, and alpha was set equal to 0.05 for statistical tests.

## Results

### RNA-Seq Analysis of Heat Shocked Mouse Utricle Explants Produces a Heat Shock Response Transcriptional Signature

**FIGURE 1:**
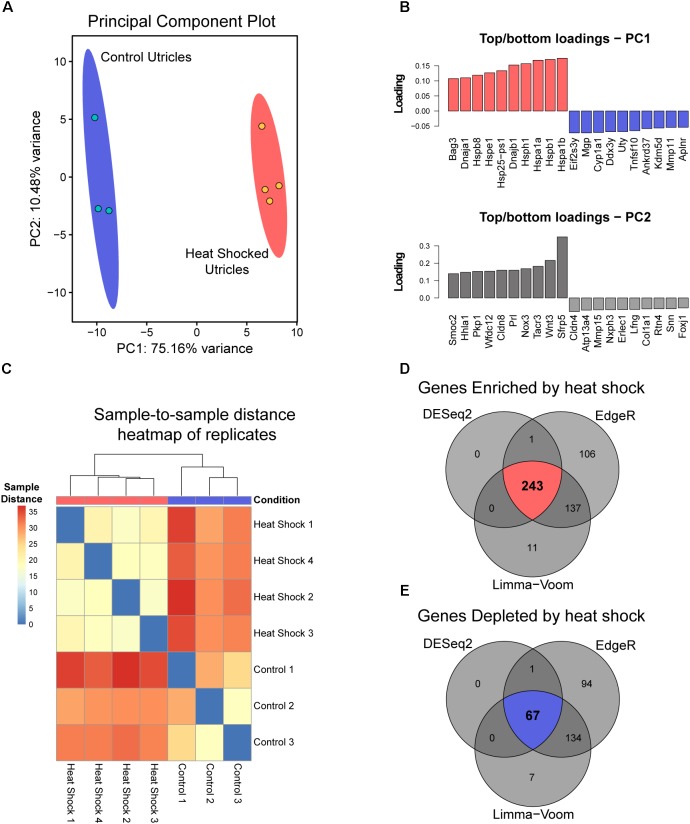
Bioinformatic analyses of whole utricle RNA-Seq data. **(A)** PCA analysis of replicates from both heat shocked (red) and control (blue) utricles using the 500 most variable genes in the dataset. PC1 represents 75.16% of the total variance in the experimental data, and PC2 represents 10.48% of the total variance. The red (heat shock) and blue (control) ellipses around each set of replicates represent 95% confidence intervals. **(B)** Top and bottom ten transcripts that contribute to PC1 and PC2 with PCA loading values plotted for each gene. **(C)** Sample-to-sample distance heatmap for RNA-Seq reads with dendrograms showing hierarchical clustering of samples based on heat shock (red) and control (blue) conditions, labeled in the row above the heatmap based on sample distances. **(D)** Results of overlapping DEGs using DESeq2, Limma-voom, and EdgeR tools with the criteria that the log_2_ fold change value must be ≥1 with an adjusted p-value < 0.05 for all three analysis tools. 243 DEGs were enriched in the heat shock condition relative to control (upper Venn diagram in red), and **(E)** 67 DEGs were depleted in the heat shock condition relative to control (lower Venn diagram in blue).

We reported that exposure to non-lethal heat shock is protective against neomycin-induced hair cell death in whole-organ cultures of utricles from adult mice (Cunningham and Brandon, 2006; Taleb et al., 2008). To determine the transcriptional profile induced by heat shock, control and heat shocked utricles were analyzed by RNA-Seq. Using the DESeq2 DEG analysis in PCAExplorer, the transcriptional changes induced by heat shock were identified globally using principal component analysis (PCA). The first principal component (PC1) accounts for 75.2% of the total experimental variance in the top 500 most variable genes. Control (*n* = 3) and heat shocked groups (*n* = 4) are completely separated along the PC1 axis (Figure 1A), suggesting that PC1 encompasses all the variation due to treatment group. No additional separation is contributed by PC2, which accounts for 10.5% of the variance. The top 10 genes contributing to PC1 are relevant to heat shock, including HSP genes (*Hspb1*, *Hspb8*, *Hspe1*, *Hsph1*, *Hspa1a*, *Hspa1b*, and *Dnaja1* encoding HSP27, HSP22, HSP10, HSP105, HSP70-1, HSP70-2, and HSP40-A1, respectively) consistent with the idea that PC1 separation represents induction of the heat shock response (Figure 1B). Sample-to-sample distance heatmapping of the individual heat shock and control replicates shows hierarchical clustering of individual samples according to condition, which is indicative of the heat shock treatment contributing to inter-sample correlation (Figure 1C). DGE analysis of transcripts enriched in the heat shock samples relative to the control samples selected genes that met the criteria of a fold change of 2 or greater (i.e., a log_2_ fold change ≥ 1) and an adjusted *p*-value or *q*-value (FDR-corrected *p*-value) of 0.05 or less. 243 DEGs (Figure 1D) met these selection criteria using all three analysis tools (DESeq2, Limma-voom, and EdgeR). 67 DEGs were identified as depleted in heat shock samples compared to controls (Figure 1E) using a fold change of 0.5 or less and a *q*-value of 0.05 or less with agreement across all three analysis tools. Enriched and depleted genes identified in this analysis are summarized in Table 3. Taken together, these analyses indicate that whole-organ, adult mouse utricle cultures can induce a robust transcriptional heat shock response.

**Table 3:** DEG analyses produce a transcriptional signature of genes that are enriched or depleted by heat shock.

Enriched DEGs (LFC ≥ 1.0, FDR ≤ 0.05)							
*Hspa1b*	*Gm9817*	*Pgf*	*RP23-16N14.2*	*1700007K13Rik*	*Mknk2*	*Gm22753*	*4833407H14Rik*
*Hspb1*	*Gm12346*	*Diras2*	*Usp43*	*Trdj1*	*Fam107b*	*Wt1*	*Knstrn*
*Hsp25-ps1*	*Fgf21*	*Amigo3*	*Gm12603*	*Igf2bp2*	*Gadd45g*	*Cdh1*	*P4ha2*
*Hsph1*	*Ahsa2*	*Hsp90ab1*	*Pdk4*	*Slc25a38*	*Ivl*	*Wnt7b*	*Procr*
*Hspa1a*	*Rpph1*	*Morc4*	*Dedd2*	*Mns1*	*Rad51c*	*Hcar2*	*Osbpl3*
*Dnajb1*	*Fam84a*	*ll33*	*Smco3*	*Sfn*	*Cer1*	*Tnfrsf19*	*Dnd1*
*Hspe1*	*Gm6335*	*Zscan29*	*Proca1*	*Pitpnm3*	*Baiap2*	*Arid5b*	*Rhof*
*Krt6a*	*Gm8696*	*Myodl*	*Kctd18*	*Gzmm*	*Bpifc*	*Sele*	*Nr1d1*
*Hspb8*	*Chordc1*	*Angpt2*	*Lingo3*	*Gm14005*	*Ptprn*	*Nipal4*	*Klhdc7a*
*Dnaja1*	*Stip1*	*DuspS*	*1200007C13Rik*	*Trib3*	*Sowahb*	*Lnx1*	*D330050G23Rik*
*Hspa1l*	*Cacybp*	*Lancl3*	*Tgfa*	*Cdr2*	*Fam46b*	*9330175E14Rik*	*Jdp2*
*Bag3*	*Cd83*	*Gm15459*	*Eepd1*	*Ptpn14*	*Macc1*	*Sh3bp2*	*Dusp4*
*Mc4r*	*Frem3*	*Gcnt2*	*Shb*	*Egr1*	*Fam83h*	*Ehd4*	*Fkbp4*
*Hspd1*	*Gm8337*	*Prrg4*	*F2rl1*	*4930563E18Rik*	*Gca*	*Krt80*	*Mfsd2b*
*Hspe1-ps3*	*Gml*	*UspH*	*Guca1b*	*Hhipl2*	*Bco1*	*Arc*	*Xk*
*Krt1*	*Aox1*	*Ari5c*	*Efhd2*	*Synpo2*	*Cnksr3*	*Gm7893*	*Myom2*
*Gm7816*	*Gm10382*	*Dnajb4*	*Mpzl3*	*Slc6a2*	*Ppl*	*P4ha1*	*Dkkl1*
*Gm15542*	*Serpinh1*	*Amotl2*	*Jun*	*Prkar1b*	*Ywhag*	*Gm14636*	*Edn1*
*Hspe1-ps2*	*Pmaip1*	*Cyr61*	*Srxn1*	*Ubc*	*Bend4*	*Mum1l1*	*Filip1l*
*Gm12141*	*Gm8326*	*Gm8428*	*Gm6368*	*Apobr*	*Dusp2*	*Gm12352*	*Vaultrc5*
*Dnaja4*	*Xirp1*	*Fam83g*	*Phlda2*	*Gm4262*	*Pde3b*	*Osmr*	*Prkab2*
*Gm26825*	*Parm1*	*inpp5j*	*RP24-282D16.4*	*Snai3*	*Pdzd2*	*Dennd1c*	
*Gm8355*	*Zfand2a*	*Gm5511*	*RP23-21P10.1*	*Baiap2l1*	*Banp*	*Msl3l2*	
*Hsp90aa1*	*5830416P10Rik*	*KcnkS*	*Fer1l5*	*Sapcd2*	*Gm8818*	*Pwwp2b*	
*Atf3*	*Ahsa1*	*Lor*	*Chka*	*Pkd1l1*	*RP23-346B12.4*	*Fam126a*	
*Gm5844*	*A530006G24Rik*	*Lamc2*	*Cdca2*	*Gpr75*	*Vgll3*	*Entpd3*	
*Muc13*	*Nog*	*Gm10069*	*Hspa4l*	*Opn3*	*Sh2d4a*	*Plin2*	
*Swt1*	*Gm29346*	*Sectmlb*	*Omp*	*Bhlha15*	*1700102P08Rik*	*Mthfd2*	
*Tubgcp4*	*Trim15*	*Arid5a*	*2310007B03Rik*	*Creb5*	*Tmcc3*	*Maff*	
*Chac1*	*Prickle1*	*Spsbl*	*Foxn1*	*Fhdc1*	*Dnajb2*	*Ahr*	
*Hspa8*	*Gm8141*	*RP24-210L3.2*	*Gprasp2*	*Rprl3*	*Gm22753*	*Fam46c*	
*Pzp*	*Cryab*	*Sid* 2a 7	*Gprasp1*	*Rgs1*	*Wt1*	*Nyap1*	

**Depleted DEGs (LFC ≥ 1.0, FDR ≤ 0.05)**							

*Cdh19*	*Fcgr2b*	*Mamdc2*	*Ramp2*	*Apba2*	*Col9a1*	*Snord13*	*Ankrd37*
*Kctd12b*	*Clec5a*	*Gper1*	*Zfyve28*	*Tmem88*	*ll15*	*Fcgr1*	*Mgp*
*Fhl3*	*Top2a*	*Dhh*	*Cd200r1*	*Ptafr*	*Pou3f1*	*Gjc3*	*Cyp1a1*
*Stox1*	*Tagln3*	*Plp1*	*Ndp*	*Nudt7*	*Fcgr3*	*Fam105a*	*Tnfsf10*
*Smtnl2*	*Slc30a3*	*Ms4a7*	*Arl11*	*Zfp773*	*C3ar1*	*Evi2a*	
*Tram1l1*	*Fam180a*	*Ly6c1*	*Sox18*	*Tlr13*	*Kcnj8*	*Slc25a18*	
*Adh7*	*Atoh1*	*Ahrr*	*Angptl2*	*Cd52*	*Meox2*	*Kcne3*	
*Ushbp1*	*Hpgds*	*Ccl12*	*Nxpe4*	*Mpz*	*8430408G22Rik*	*Mmp11*	
*Rab19*	*Gimap1*	*Scgb3a1*	*Gm29538*	*Acer2*	*Kcna1*	*Aplnr*	


*Shown are the 243 enriched DEGs (red) and 67 depleted DEGs (blue) identified using the overlap of DESeq2, Limma-voom, and EdgeR DEG analysis tools as shown in Figure 1D ordered left-to-right in columns by the DEG magnitude of DESeq2 log_*2*_ fold changes.*

### RT-qPCR Validation of the Heat Shock Transcriptional Signature

**FIGURE 2:**
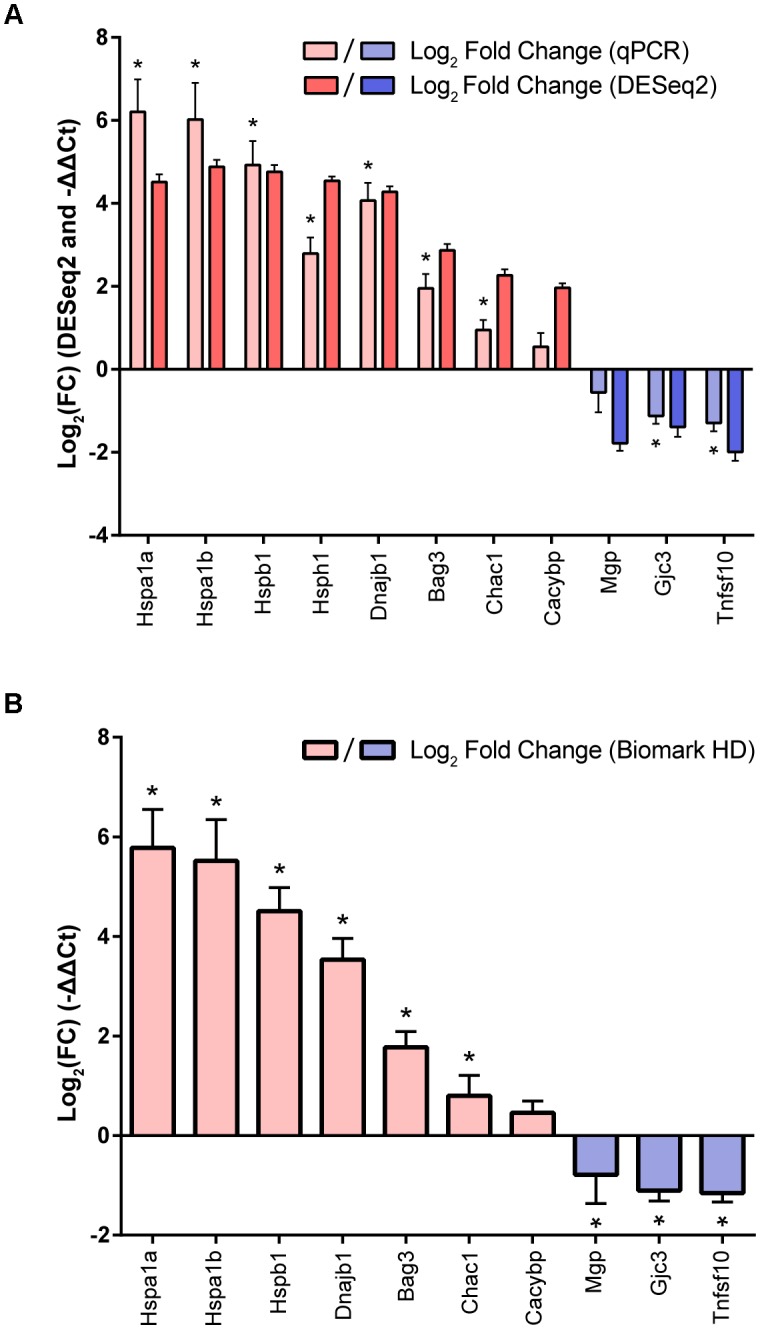
Validation of the utricle heat shock transcriptional signature. **(A)** Fold changes (mean ± standard error values calculated from the DESeq2 model) of eight enriched and three depleted DEGs from the heat shock transcriptional signature are shown in dark red and dark blue, respectively. RT-qPCR fold changes (normalized to Actb) performed in independent replicates (*n* = 3 biological replicates per group) for the same eight enriched and three depleted DEGs are shown in light red and light blue, respectively. **(B)** Fold changes from independent replicates for seven enriched and three depleted DEGs from the heat shock signature normalized to Gapdh and measured on the Biomark HD system show similar induction patterns as measured in **(A)**. Asterisks indicate statistically significant (*p* < 0.05) differences in heat shock ΔCt values compared to control replicates as measured by multiple unpaired t-tests following Holm-Sidak multiple comparison correction, represented above each ΔΔCt value.

To validate the signature of enriched and depleted DEGs, eight genes (*Hspa1a, Hspa1b, Hspb1, Hsph1, Dnajb1, Bag3, Chac1, Cacybp*) were selected from the enriched DEG set at approximately the 80th percentile or above (ranging from the ∼83rd percentile for *Cacybp* to the 100th percentile for *Hspa1b*, ordered by DESeq2 log_2_ fold change), and three genes (*Mgp, Tnfsf10, Gjc3*) were selected from the depleted DEG set at the 15th percentile or lower (ranging from the 0th percentile for *Tnfsf10* to the ∼15th percentile for *Gjc3*, ordered by DESeq2 log_2_ fold change) for qPCR analysis using TaqMan assays. Independent samples of cultured utricles (*n* = 3 per condition) were prepared as heat shock or control as in the RNA-Seq experiment, and qPCR was performed on the total RNA from these samples (Figure 2A). The gene expression patterns identified by RNA-Seq analysis were reproducible in this independent experiment (normalized to *Actb*), with 7/8 genes from the enriched DEG set (*Hspa1a, Hspa1b, Hspb1, Hsph1, Dnajb1, Bag3, Chac1*) significantly induced after heat shock, and 2/3 genes from the depleted DEG set (*Gjc3*, *Tnfsf10*) were significantly depleted. Two genes (*Cacybp* from the enriched DEG set and *Mgp* from the depleted DEG set) did not reach statistical significance after multiple corrections, but enrichment (ΔΔ*C*t = 0.54 ± 0.33, *p*-value = 0.10) or depletion (ΔΔ*C*t = -0.56 ± 0.48, *p*-value = 0.089) in the predicted directions did occur, respectively, in these genes relative to the control (no heat shock group). Although the log_2_FC values from DESeq2 and the log_2_(ΔΔ*C*t) values are not directly statistically comparable because measurements were made in different sample sets using different normalization methods, the Pearson correlation coefficient for the log-transformed fold changes for all 11 validation genes was equal to 0.91 (*p* < 0.0001) between the Taqman qPCR ΔΔCt measurements and DESeq2 fold change measurements, indicative of significant correlation between the two independent sets of gene expression patterns as a result of heat shock.

Changes in gene expression between heat shock and control replicates were also validated for 10 validation genes on a Fluidigm Biomark HD platform (Figure 2B). Expression of 6/7 genes (*Hspa1a, Hspa1b, Hspb1, Dnajb1, Bag3, Chac1*) from the enriched DEG set were significantly induced. Expression of 3/3 genes (*Mgp, Gjc3, Tnfsf10*) were significantly reduced. Again, changes in *Cacybp* were found to be not statistically significant. Reduction of *Mgp* expression was statistically significant on the Biomark HD platform, in contrast to the TaqMan analysis, suggesting a borderline significance of this result in concordance with the RNA-Seq DEG results. Furthermore, ΔΔ*C*t values of all 10 genes calculated using both the Applied Biosystems and the Biomark HD platforms were highly correlated, with a Pearson correlation coefficient of 0.99 (*p* < 0.0001), indicating high levels of agreement between the two sets of measurements. Together our validation experiments using two different methods confirm the overall pattern of upregulated and downregulated genes we observed in the RNA-Seq dataset.

### LINCS Query of the Heat Shock Gene Expression Signature Provides a Ranking of Small Molecule Perturbagens That Produce Similar and Opposite Transcriptional Profiles in Tested Cell Lines

**FIGURE 3:**
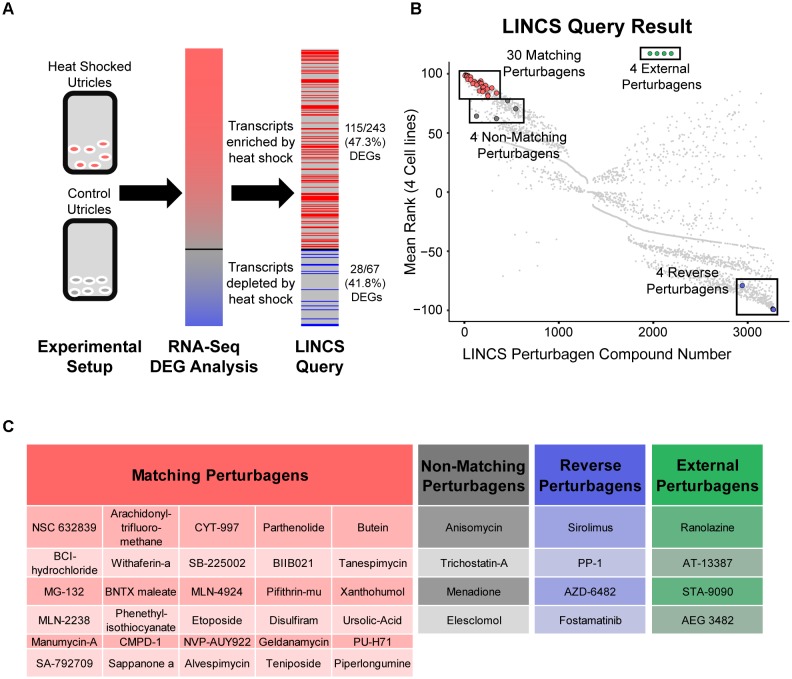
LINCS identification of small molecule perturbagens that mimic the heat shock transcriptional signature. **(A)** Schematic of the LINCS workflow. Utricle cultures were analyzed by RNA-Seq to obtain a DEG signature of transcripts enriched or depleted by heat shock compared to control (red/blue gradient bar representing DEGs in Figure 1D and Table 1). The DEG signature was used as input for the LINCS Query tool, which recognized 115/243 (47.3%) enriched DEGs and 28/67 (41.8%) depleted DEGs. **(B)** Scatterplot of the 3,273 small molecule perturbagen signatures in the LINCS Query database showing the normalized rank match in four core LINCS cell lines compared to the input signature from heat shocked utricles. A subset of the LINCS-identified perturbagens was selected and screened for otoprotection and induction of the heat shock DEG signature. Thirty matching perturbagens (shown in red) were selected from the ≥90th percentile of all LINCS signatures that matched the input DEG signature of heat shocked utricles. Four perturbagens (dark gray) between the 80th and 90th percentiles were selected as non-matching perturbagens. Four perturbagens (blue) that yielded the opposite signature to the input DEG signature were chosen as reverse perturbagens. Four compounds (green) chemically similar to LINCS-identified compounds were added as external comparisons to the LINCS dataset. **(C)** The 30 matching perturbagen compound names are in red. The four non-matching perturbagen names are shown in gray; the four reverse perturbagen names are in blue, and the four external perturbagen names are in green.

We used LINCS analysis to compare signatures of DEGs found after heat shock to a database of gene expression changes found after treatment with a perturbagen drug library. The iteration of the LINCS tool used in this analysis (Subramanian et al., 2017) accepted lists of enriched and depleted genes without fold change information. The LINCS L1000 assay measured 987 landmark genes and then computationally inferred the expression level of a total of approximately 11K genes (Duan et al., 2014; Subramanian et al., 2017). As a consequence, the query tool recognized only a subset of the genes entered from our RNA-Seq experiment: The LINCS query tool recognized 115/243 (47.3%) transcripts in the enriched DEGs, and 28/67 (41.8%) transcripts in the depleted DEGs (Figure 3A) for a total of 143/310 (46.1%) of the DEGs from our RNA-Seq dataset. The LINCS tool then ranked 3,273 perturbagen signatures according to how closely they induced gene expression changes that matched the input DEG subset from our heat shock RNA-Seq data. The ranking for each perturbagen signature was the normalized average rank match from four separate cell lines tested with each perturbagen in LINCS (Figure 3B). ‘Matching’ perturbagens are those that produce gene expression changes similar to our input DEG list of transcripts enriched/depleted by heat shock (≥90th percentile mean rank). ‘Reverse’ perturbagens are those that produce gene expression changes that are opposite from our input list (≤10th percentile mean rank). Our analysis revealed 328 matching and reverse perturbagen signatures using these cut offs. Small molecules that were chosen for analysis from outside of the LINCS dataset we refer to as ‘external’ perturbagens (Figure 3B).

We selected a subset of perturbagens to test whether the protective effect of heat shock in hair cells could be reproduced (or reversed) by a small molecule. Thirty matching perturbagens (Figures 3B,C, red) and four reverse perturbagens (Figures 3B,C, blue) were chosen for further analysis. In addition, four ‘non-matching’ perturbagens were chosen from below the 90th percentile LINCS ranking (Figures 3B,C, gray). Four ‘external’ perturbagen molecules related to matching perturbagens but not found in the LINCS analysis were also selected for screening (Figures 3B,C, green). Thus, the LINCS query tool allowed us to design a targeted list of compounds capable of either matching or inverting our input gene expression signature.

### Matching LINCS Perturbagens Share Drug Classifications and Have Precedent in Existing Literature for Hair Cell Protection Against Ototoxic Drugs

The LINCS-generated list of matching perturbagens included some classes of drugs that are known to protect hair cells. The molecular targets and signature rankings for each perturbagen in the screen are listed in Table 4. Six out of the 30 matching LINCS perturbagens are HSP90 inhibitors (NVP-AUY922, Alvespimycin, BIIB021, Geldanamycin, Tanespimycin, PU-H71), which is not surprising given that HSP90 inhibition results in transcriptional activation of HSPs via the transcription factor Hsf1 (Whitesell et al., 2003). Two members of the HSP90 inhibitor group, Alvespimycin and Geldanamycin, are protective against aminoglycoside-induced hair cell death (Yu et al., 2009; Liu Y. et al., 2015) and can induce expression of HSPs in inner ear tissue. For this reason, we selected two additional HSP90 inhibitors (AT-13387, STA-9090) to examine as well as another known HSP inducer, AEG 3482 (Salehi et al., 2006). Some additional compounds from the LINCS matching list were either known otoprotectants or were chemically related to known otoprotectants. These included proteasome inhibitors MG-132 and MLN-2238, the mitochondrial p53/HSP70 inhibitor pifithrin-μ, and the anti-inflammatory compound piperlongumine (Coffin et al., 2013a,b; Yadav et al., 2014). Thus, the results of the LINCS query are supported by literature indicating that several matching perturbagens are known to be protective in the inner ear.

**Table 4:** A detailed list of the 42 compounds chosen for otoprotection screening identified in Figure 3B.

Drug name	Description	Rank score
NSC 632839	Ubiquitin isopeptidase inhibitor	99.1
BCI-hydrochloride	MAP kinase phosphatase (Dusp6) inhibitor	98.9
MG-132	Proteasome inhibitor	98.8
MLN-2238	Proteasome inhibitor	98.8
Manumycin-A	Farnesyltransferase inhibitor, NF-kB pathway inhibitor	98.4
SA-792709	Retinoid receptor gamma agonist	98.2
Arachidonyl-trifluoro-methane	Cytosolic phospholipase inhibitor	97.9
Withaferin-a	Acetylcholinesterase inhibitor, butyrylcholinesterase inhibitor, IKK inhibitor, NF-kB pathway inhibitor, PKC inhibitor	97.3
BNTX maleate	δ1 opioid receptor antagonist	97.2
Phenethyl-isothiocyanate	Cancer cell growth inhibitor	95.2
CMPD-1	MAPKAPK2 inhibitor	94.9
Sappanone a	Hsf1 inducer	94
CYT-997	Tubulin polymerization inhibitor	93.2
SB-225002	CXCR2 chemokine receptor antagonist	92.5
MLN-4924	NEDD activating enzyme inhibitor	92.1
Etoposide	Topoisomerase inhibitor	92.1
NVP-AUY922	HSP90 inhibitor	91.7
Alvespimycin	HSP90 inhibitor	91.6
Parthenolide	NF-kB pathway inhibitor, adiponectin receptor agonist	90.6
BIIB021	HSP90 inhibitor	90
Pifithrin-μ	HSP70 inhibitor	89.2
Disulfiram	Aldehyde dehydrogenase inhibitor, DNA methyltransferase inhibitor, TRPA1 agonist	88.4
Geldanamycin	HSP90 inhibitor	88.1
Teniposide	Topoisomerase inhibitor, DNA repair enzyme inhibitor, mitotic inhibitor	87.1
Butein	Angiotensin converting enzyme inhibitor, epidermal growth factor receptor (EGFR) inhibitor	85.6
Tanespimycin	HSP90 inhibitor	85.5
Xanthohumol	Aromatase inhibitor, diacylglycerol *O*-acyltransferase inhibitor, valosin containing protein inhibitor	84.9
Ursolic-acid	Antioxidant/general anti-inflammatory	83.7
PU-H71	HSP90 inhibitor	82.2
Piperlongumine	Glutathione transferase inhibitor	81.2
Anisomycin	DNA synthesis inhibitor	77.2
Trichostatin-A	HDAC inhibitor, CDK expression enhancer, ID1 expression inhibitor	70.4
Menadione	CDC inhibitor, mitochondrial DNA polymerase inhibitor	64.2
Elesclomol	Apoptosis stimulant, HSP agonist, HSP inducer, oxidative stress inducer, topoisomerase inhibitor	62.1
Sirolimus	mTOR inhibitor	–79.1
PP-1	Arc inhibitor, Abl kinase inhibitor	–988
AZD-6482	PI3K inhibitor	–99.2
Fostamatinib	Syk inhibitor, FLT3 inhibitor	–99.4
Ranolazine	Fatty acid oxidation partial inhibitor, sodium channel blocker	External
AT-13387	HSP90 inhibitor	External
STA-9090	HSP90 inhibitor	External
AEG 3482	HSP inducer, JNK inhibitor	External


*The compound names, putative molecular targets or functions and the mean normalized rank in four cell lines compared to the input DEG signature are shown in separate columns. The compounds are color-coded to show matching perturbagens in red, non-matching perturbagens in gray, reverse perturbagens in blue, and external perturbagens in green.*

### Screening Selected LINCS Perturbagens in Zebrafish Against Neomycin-Induced Ototoxicity Yielded Three Otoprotective Hits

**FIGURE 4:**
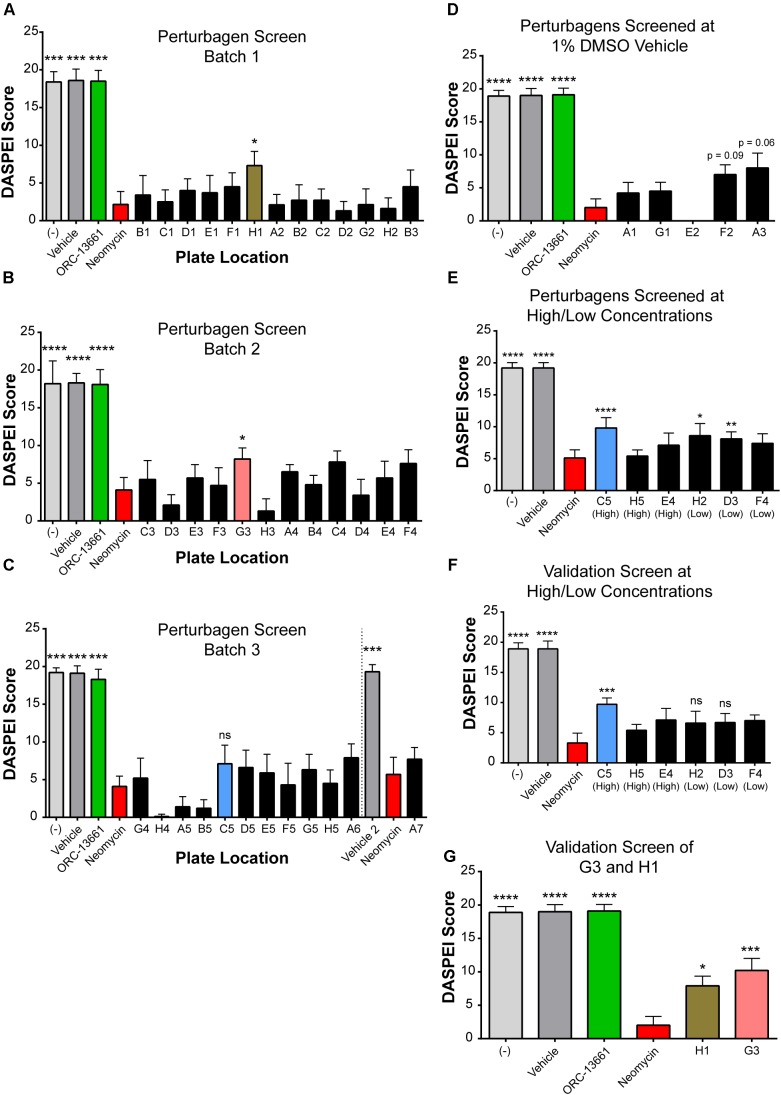
LINCS-identified perturbagen screening in zebrafish revealed three compounds that protect against neomycin-induced hair cell death in lateral line neuromasts. **(A–D)** DASPEI screening of perturbagens against neomycin-induced ototoxicity in zebrafish neuromasts. Negative controls (–) are shown in light gray (*n* = 5–10 zebrafish per bar); vehicle controls (0.1–1% DMSO or 0.1% ethanol) are shown in gray (*n* = 5–10), and positive controls with the otoprotective compound ORC-13661 are shown in green (*n* = 5–10). 200 μM neomycin (red) resulted in significant hair cell death (*n* = 10–20). Perturbagens that were either not protective or were protective but failed to replicate in a validation experiment are shown in dark gray. Three perturbagens, designated according to their locations on the culture plate as C5 (blue), G3 (pink), and H1 (brown), were protective against neomycin-induced hair cell death in this screen (*n* = 10 for all perturbagen treatment bars). **(A)** DASPEI scores from the first screen of perturbagens tested at 10 μM against 200 μM neomycin. H1 (AT-13387, brown) was protective in this batch. **(B)** DASPEI scores from the second batch of perturbagens screened at 10 μM against 200 μM neomycin. G3 (Pifithrin-μ, pink) was identified as a protective hit in this batch. **(C)** Third batch of compounds screened at 10 μM against 200 μM neomycin. C5 (AEG 3482, blue) was later identified as a hit at a different concentration but was not significantly protective at 10 μM (*p* > 0.05). **(D)** Remaining batch of perturbagens screened at 1% DMSO and 10 μM concentration to increase solubility. **(E)** DASPEI scores using a batch of compounds at alternative high or low doses. C5 was identified as a hit at 30 μM. **(F)** DASPEI scores from the C5 validation screen of alternative high or low dose compounds. **(G)** DASPEI scores from the validation experiment for 10 μM H1 and 10 μM G3 hits demonstrating a repeatable protective effect against 200 μM neomycin. Asterisks represent adjusted p-values for Dunn’s multiple comparisons test following a Kruskal–Wallis test for treatment with ^∗^ representing *p* ≤ 0.05, ^∗∗^ representing *p* ≤ 0.01, ^∗∗∗^ representing *p* < 0.001, ^∗∗∗∗^ representing *p* < 0.0001, and ‘ns’ (not significant) representing *p* > 0.05. All error bars in **(A–G)** represent ± SD values.

Based on the results above, we screened the 42 LINCS-identified perturbagens and related compounds (Figure 3C) for their effects on aminoglycoside-induced hair cell death in the zebrafish lateral line. Zebrafish larvae (*n* = 10 larvae per perturbagen treatment and *n* = 5–10 larvae for control groups, aged 5–7 dpf) were exposed to neomycin (200 μM) for 1 h in the presence or absence of each perturbagen (10 μM). Ten neuromasts on each fish were scored (ranging between 0 and 2 per neuromast) based on the observer’s estimate of the fluorescence intensity of the DASPEI label (Harris et al., 2003) (see the section “Materials and Methods”). Screening was performed in batches, with each 48-well plate including its own positive and negative controls (Figure 4). The previously identified otoprotective compound ORC-13661 (10 μM) (Chowdhury et al., 2018), used as a positive control, demonstrated consistent significant protection from 200 μM neomycin (Figures 4A–D,G, green bars). The criteria for a compound to be called protective in the initial screen was to achieve statistical significance (*p* < 0.05) as compared to the neomycin alone group based on the DASPEI score. The significant protective effect had to then be replicated in a secondary validation screen. Some compounds look quite close in terms of DASPEI score in the initial screen (Figures 4A–C) but were discarded after failing validation in a secondary screen. Protective “hits” identified in the initial perturbagen screen were AT13387 (plate location H1) (Figure 4A, brown bar, *p* = 0.01) and Pifithrin-μ (G3) (Figure 4B, pink bar, *p* = 0.048). The protective effects of each of these compounds were then validated in a replication experiment (Figure 4G, *p* = 0.04 and 0.0009, respectively). AEG 3482 (C5) (Figure 4C, blue bar) was not significantly protective at 10 μM. However, a follow-up round of screening was performed at alternative doses for some compounds based on specificity for the molecular targets and compound activities listed from vendors. AEG 3482 has a reported EC50 value of 20 μM in the prevention of neuronal death caused by nerve growth factor (NGF) withdrawal (Salehi et al., 2006), so a high concentration (30 μM) was attempted in the DASPEI assay (Figure 4E, blue) and was found to be significantly protective (*p* = 0.0003). The protective effect of AEG 3482 was then replicated in a subsequent experiment (Figure 4F). Certain highly specific compounds (MG-132, MLN-2238, PU-H71) were also screened at a lower concentration (1 μM), and results from the either high or low dose were then validated. MG-132 (D3) and MLN-2238 (H2) were each significantly protective in the first round of the low-dose screen (Figure 4E, *p* = 0.02, and *p* = 0.0075, respectively); however, the protective effect of these two perturbagens was not replicated in the validation screen (Figure 4F, *p* = 0.33 and 0.32, respectively) and so they were not examined further.

**FIGURE 5:**
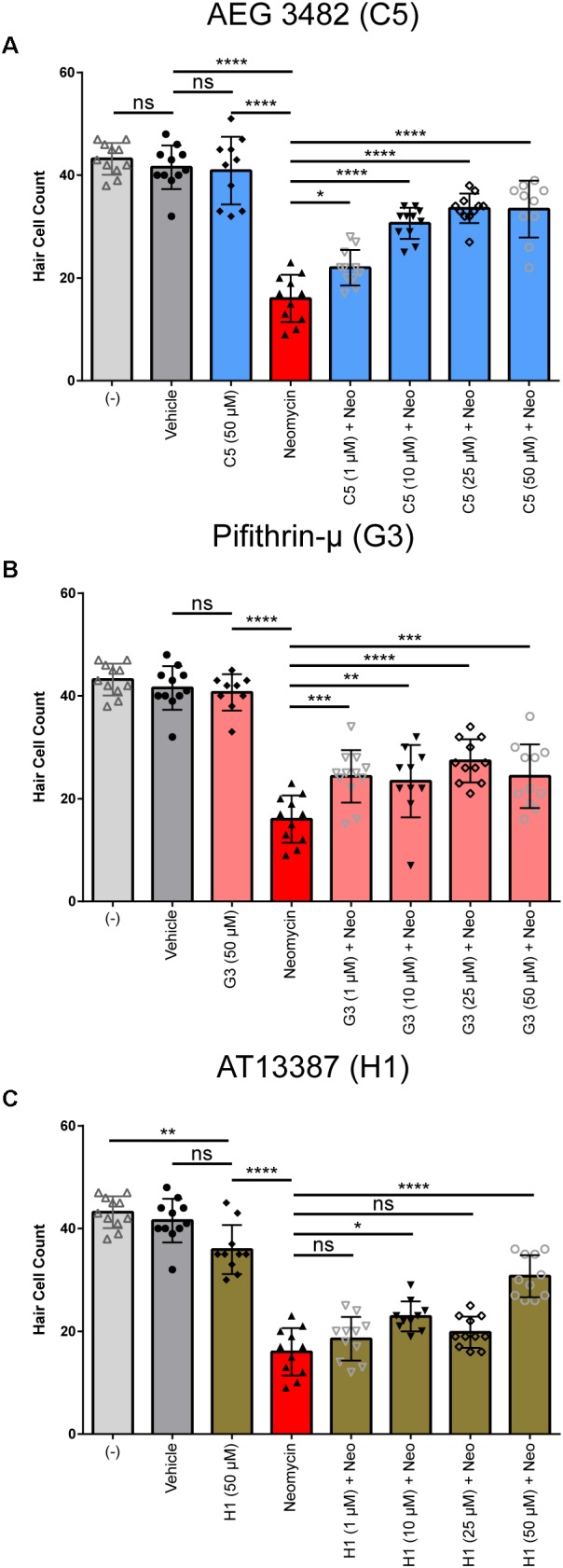
All perturbagen hits show protection against neomycin-induced hair cell death at multiple doses. **(A)** Dose–response relationship for C5 (AEG 3482, blue). Hair cells were counted from anti-parvalbumin-labeled neuromasts. **(B)** Dose–response relationship for G3 (Pifithrin-μ, pink), **(C)** Dose–response relationship for H1 (AT-13387, brown). Negative control, vehicle, and neomycin plus vehicle groups are identical in all three experiments, as all doses for all three drugs were tested in the same experiment (*n* = 8–10 per group) but are stratified into separate graphs for clarity of the comparisons being made. Asterisks represent adjusted p-values from Sidak multiple comparisons test following one-way ANOVA with ^∗^ representing *p* ≤ 0.05, ^∗∗^ representing *p* ≤ 0.01, ^∗∗∗^ representing *p* < 0.001, ^∗∗∗∗^ representing *p* < 0.0001, and ‘ns’ (not significant) representing *p* > 0.05. All error bars in **(A–C)** represent ± SD values.

Following identification and validation of the three perturbagen hits (AT13387, Pifithrin-μ, AEG 3482) in the DASPEI screen, we examined the dose–response relationship of each of these compounds. Each compound was tested at 1, 10, 25, and 50 μM as co-treatment with 200 μM neomycin for 1 h (*n* = 9–11 larvae per treatment group). The highest concentration (50 μM) for each perturbagen was tested without neomycin to evaluate its potential toxicity. Treatment with AEG 3482 showed a clear dose–response relationship, with survival of hair cells significantly increasing with increasing concentrations of AEG 3482 with neomycin compared to neomycin alone (Figure 5A, blue bars, *p* < 0.05 at 1 μM, *p* < 0.0001 at 5, 10, 25, and 50 μM). Treatment with Pifithrin-μ did not show a dose–response relationship, but rather demonstrated significant protection against neomycin at all doses tested (Figure 5B, pink bars, *p* < 0.001 at 1 μM, *p* < 0.01 at 5 μM, *p* < 0.0001 at 10 μM, *p* < 0.001 at 50 μM). AT13387 treatment resulted in an inconsistent protective effect, with significant protection observed at the 10 μM and 50 μM doses (Figure 5C, brown bars, *p* = 0.0033 at 10 μM and *p* < 0.0001 at 50 μM) but not at 25 μM. The results of the DASPEI screen and dose–response assays indicate that AEG 3482, Pifithrin-μ, and AT13387 can protect against neomycin-induced hair cell death in zebrafish lateral line neuromasts.

### LINCS Hits Induce the Heat Shock Transcriptional Expression Signature in Cultured Mouse Utricles

**FIGURE 6:**
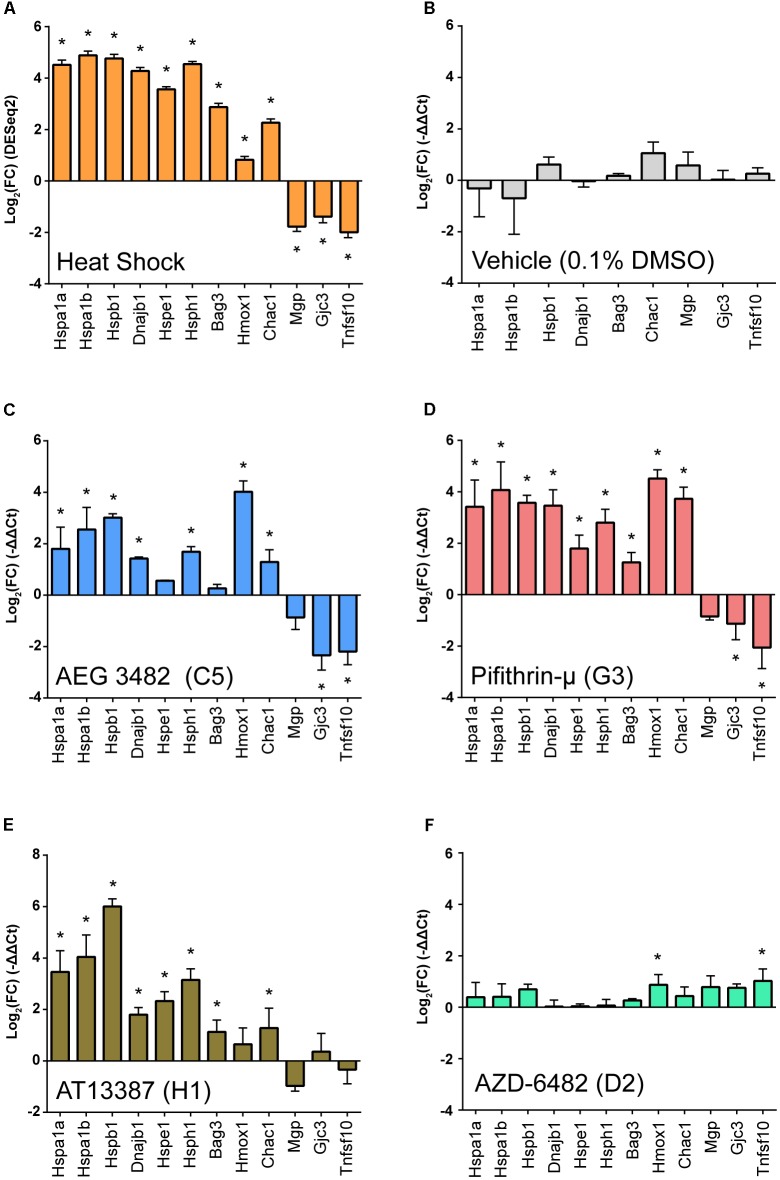
The perturbagen hits induce the heat shock transcriptional signature in cultured mouse utricles. Gene signature profiling was performed using Taqman qPCR assays. **(A)** The heat shock transcriptional profile. Shown are log_2_ fold changes (from DESeq2 DEG analysis) for heat shocked utricles compared to controls. Asterisks indicate significance, and error bars are ± SE for each log_2_ fold change. **(B)** The vehicle used for the perturbagens does not induce a heat shock transcriptional signature. Shown are log_2_ fold changes (ΔΔCt) for genes measured in utricles exposed to 0.1% DMSO vehicle compared to non-heat shocked control utricles normalized to Gapdh using the Biomark HD platform. **(C–F)** Examination of the transcriptional signatures induced by three perturbagen hits and one reverse hit. Gene expression was measured in seven enriched and three depleted DEGs from the RNA-Seq DEG signature validation in Figure 2 in addition to two additional genes, Hmox1 and Hspe1 on the Applied Biosystems platform. **(C)** AEG 3482 induces the heat shock transcriptional signature in utricles. Shown are log_2_ fold changes (ΔΔCt) in utricles treated with AEG3482 (25 μM) normalized to Actb. **(D)** Pifithrin-μ induces the heat shock transcriptional signature in utricles. Shown are log_2_ fold changes (ΔΔCt) in utricles treated with pifithrin-μ compared to vehicle. **(E)** AT-13387 induces the heat shock transcriptional signature in utricles. Shown are log_2_ fold changes (ΔΔCt) in utricles treated with AT-13387 compared to vehicle. **(F)** The reverse perturbagen AZD-6482 does not induce the heat shock transcriptional signature. Shown are log_2_ fold changes in utricles treated with AZD-6482 compared to vehicle. Asterisks in **(A–D)** indicate significant (*p* < 0.05) ΔCt differences compared to DMSO vehicle ΔCt values in multiple unpaired *t*-tests following Holm-Sidak multiple comparison correction (*n* = 3 biological replicates per group).

In order to examine the transcriptional response of the three protective compounds in a mammalian system, we applied each compound to whole organ cultures of utricles from adult mice (Brandon et al., 2012). Seven of the eight transcripts validated by qPCR analysis that were increased after heat shock (*Hspa1a, Hspa1a, Hspb1, Dnajb1, Hsph1, Bag3, Chac1*) were examined with an additional two enriched genes from the RNA-Seq data (*Hspe1* and *Hmox1*), and three of the transcripts validated by qPCR that were depleted after heat shock (*Mgp*, *Gcj3*, *Tnfsf10*) were measured after perturbagen exposure. Each perturbagen was administered as a 6-h pre-treatment followed by immediate RNA extraction. DESeq2 fold changes are shown in Figure 6A. No significant changes in expression of heat shock signature genes was noted in utricles treated with vehicle alone (0.1% DMSO) (Figure 6B). Treatment with AEG 3482 (Figure 6C) resulted in significant induction of 7/9 transcripts in the heat shock transcriptional signature (*Hspa1a, Hspa1b, Hspb1, Dnajb1, Hsph1, Hmox1, Chac1; p* < 0.05, multiple unpaired two-tailed *t*-tests with Holm-Sidak multiple comparisons adjustment) and significantly reduced expression of 2/3 heat shock-depleted transcripts (*Gjc3, Tnfsf10*). Pifithrin-μ (Figure 6D) significantly induced 9/9 heat shock-enriched transcripts and significantly reduced 2/3 heat shock-depleted transcripts (*Gjc3*, *Tnfsf10*). AT13387 significantly induced expression of 8/9 heat shock-induced transcripts (*Hspa1a, Hspa1b, Dnajb1, Hspb1, Hspe1, Hsph1, Bag3, Chac1*) and reduced expression of 0/3 heat shock-depleted transcripts (Figure 6E). The gene expression profile of AZD-6482, one of the LINCS reverse perturbagens, (i.e., in the bottom 10th percentile of LINCS-identified perturbagens), (Figure 6F) induced expression of only 1/9 heat shock-induced transcripts genes (*Hmox1*), and significantly induced expression of 1/3 heat shock-depleted transcripts (*Tnfsf10*). Individual fold-change magnitudes differed for each perturbagen. For example, *Hmox1* enrichment differed substantially, with AEG-3482 and Pifithrin-μ exposures resulting in significant and considerable *Hmox1* induction (roughly 16-fold and 23-fold induction compared to vehicle treatment, respectively), whereas AT13387 induced only modest *Hmox1* expression (roughly 1.6-fold) compared to vehicle treatment. No direct statistical comparison between qPCR gene expression patterns from each perturbagen treatment can be made, due to differences in vehicle-treated samples and inter-plate variability; however, we observed significant positive Pearson correlation coefficients between heat shock and AEG 3482 (0.71, *p* = 0.009), Pifithrin-μ (0.80, *p* = 0.002), and AT13387 (0.86, *p* = 0.0003) treatments. A significant negative Pearson correlation coefficient was observed between heat shock and AZD-6482 (-0.74, *p* = 0.006) treatment, and there was no significant correlation between vehicle treatment and heat shock (-0.33, *p* = 0.38), AEG 3482 (-0.14, *p* = 0.72), Pifithrin-μ (-0.15, *p* = 0.69), AT13387 (-0.26, *p* = 0.50), or AZD-6482 (0.29, *p* = 0.44) treatments (See Supplementary Figure S1). Taken together, these data indicate that each perturbagen hit that was protective in zebrafish induced a gene expression signature in mouse utricle that resembled the heat shock transcriptional signature.

### The Perturbagen Pifithrin-μ Reduces Neomycin-Induced Hair Cell Death in Cultured Utricles From Adult Mice

**FIGURE 7:**
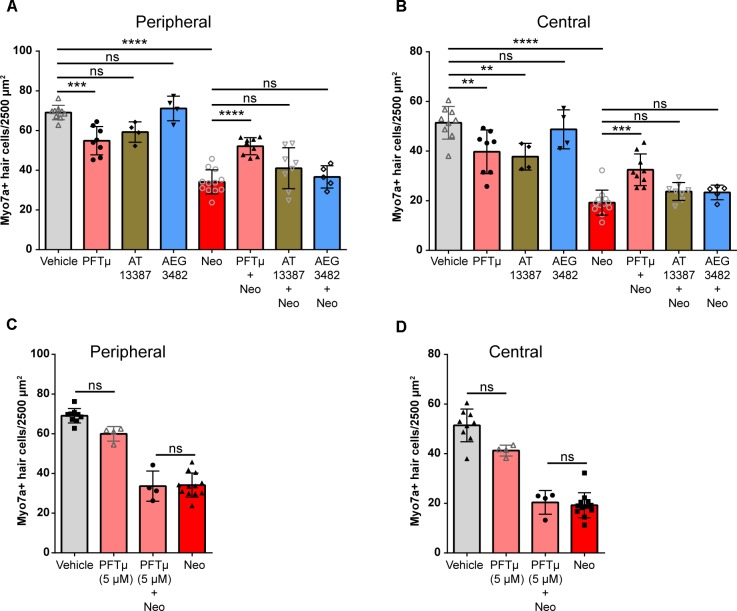
Perturbagen hit pifithrin-μ reduces aminoglycoside-induced hair cell death in cultured mouse utricles. **(A)** Myosin VIIa- and Hoechst 33342-labeled hair cell counts in the peripheral region of utricles treated with vehicle (0.1% DMSO, gray), pifithrin-μ (10 μM, pink), AT13387 (10 μM, brown), or AEG3482 (25 μM, blue). **(B)** Hair cell counts labeled as in **(A)** for the central region of the utricle. Hair cell count results in **(A–D)** are pooled from two independent experiments with the significant effects replicated in the second experiment (*n* = 4–12 utricles per group). **(C,D)** Reducing the concentration of pifithrin-μ to 5 μM reduces the toxicity of the compound but also abolishes the protection against neomycin-induced hair cell death in both the peripheral **(C)** and central **(D)** regions of the utricle. Asterisks represent adjusted *p*-values from Tukey *post hoc* test results following one-way ANOVA with ^∗∗∗^ representing *p* < 0.001, ^∗∗∗∗^ representing *p* < 0.0001, and ‘ns’ (not significant) representing *p* > 0.05. All error bars in **(A–D)** represent ± SD values.

We next examined whether the three protective perturbagens identified in the zebrafish screen reduce aminoglycoside-induced hair cell death in mouse utricles (*n* = 4–12 utricles per treatment from two to six mice). AEG 3482 was not protective against neomycin-induced hair cell death in either the peripheral or central regions of the utricle (Figures 7A,B, blue bars). AT13387 alone was toxic to hair cells, reducing survival in the central region but not in the peripheral region (Figures 7A,B, brown bars). AT13387 was not protective in either region. In the absence of neomycin, pifithrin-μ (Figures 7A,B, pink bars) caused a significant reduction in the number of hair cells in both regions compared to vehicle alone; however, pifithrin-μ was also significantly protective against neomycin-induced hair cell death (Figures 7A,B, pink bars). Of the three perturbagen hits that were protective in the zebrafish screen, only pifithrin-μ was protective against neomycin-induced hair cell death, although it also independently caused some damage to hair cells as a single treatment. To assess whether reducing the dose of pifithrin-μ would reduce the toxic effect we observed, we also tested pifithrin-μ at 5 μM, which eliminated both the toxicity and the protective effect of the compound (Figures 7C,D) (*p* > 0.99 for both comparisons). Overall our data indicate that the LINCS tool generated a list of perturbagens that matched the transcriptional profile of protective heat shock. We screened 43 perturbagens in zebrafish, and 3 (∼7%) of these were protective. One of these, pifithrin-μ, was also protective in a mammalian inner ear system.

## Discussion

This study utilized a workflow that began with a previously identified otoprotective stimulus, heat shock, and ended with a small molecule that could recapitulate both the gene expression profile and otoprotective effect of heat shock. The LINCS database was used to identify perturbagens that match the transcriptional signature of heat shock, which were then moved through two separate model systems to test effects on inner ear ototoxicity: The zebrafish neuromast and the adult mouse utricle. Pifithrin-μ was identified as a perturbagen that recapitulates both the gene expression profile and protective effect of heat shock.

We can make several observations from this workflow pertaining to the use of LINCS, a bioinformatic tool that allowed us to move from an RNA-Seq gene expression profile into a perturbagen screening assay by matching the heat shock gene expression pattern to a database of gene expression patterns in cell lines exposed to small molecules. Using the results from the LINCS Query alone, only a subset of the genes that were used as inputs are recognized by the L1000 gene expression assay used in the LINCs database. In addition, there is a lack of fold enrichment/depletion information for use in this query, which reduces the complexity of the gene expression pattern into a binary (induced/depleted) comparison. In addition, the LINCS query expression patterns are made against nine core human cancer cell lines, which may respond to perturbagen application very differently from inner ear epithelia. Despite these limitations, the perturbagens returned from LINCS query using the heat shock signature did recapitulate the heat shock gene expression signature in the cultured utricle system. Because only the perturbagens that were hits in the zebrafish screen were carried forward into utricle model, we cannot conclude that every matching perturbagen would match the heat shock signature; however, we can say that AT13387, being an HSP90 inhibitor and external perturbagen, did induce the expected heat shock expression signature. We also tested the expression pattern of cultured utricles exposed to reverse perturbagen AZD-6482, and this exposure resulted in a significantly DGE pattern by qPCR compared to the matching perturbagen pifithrin-μ, suggesting that the LINCS designation of ‘matching’ versus ‘reverse’ provides specificity in gene expression patterning despite some limitations of the query tool.

Our LINCS query results include several compounds that have been previously investigated with respect to ototoxicity. The HSP90 inhibitor Alvespimycin (also known as 17-DMAG) has been shown to protect against kanamycin exposure in mouse neonatal cochlear explants and to induce HSP70 localized by immunohistochemistry to inner and outer cochlear hair cells (Liu Y. et al., 2015). Geldanamycin, another HSP90 inhibitor, was effective in reducing gentamicin-induced hair cell death in organ of Corti explants (Yu et al., 2009). Pifithrin-μ was previously found to be protective against neomycin- and gentamicin-induced damage in zebrafish neuromasts (Coffin et al., 2013a). The proteasome inhibitors MLN-2238 and MG-132 are related to Z-LLF-CHO, a proteasome inhibitor that protects against gentamicin, neomycin, and cisplatin-induced ototoxicity in zebrafish (Coffin et al., 2013b). Etoposide and teniposide, both inhibitors of topoisomerase 2, share molecular target activity with amsacrine, an antineoplastic agent with topoisomerase II inhibition activity that is otoprotective against aminoglycoside-induced hair cell death in the zebrafish lateral line (Ou et al., 2009). In the ‘non-matching perturbagen’ category, the perturbagen Trichostatin A (LINCS query signature 86th percentile), a class I/II HDAC inhibitor, protects early postnatal organ of Corti explants against cisplatin-induced ototoxicity *in vitro* and also induced the expression of several genes related to synaptic plasticity that had been downregulated by cisplatin exposure (Wang et al., 2013). Parthenolide (LINCS query signature 95th percentile), an NF-κB inhibitor, increased apoptotic signaling in rat cochlear explants and synthetic peptide inhibition of NF-κB induced significant hair cell death in these explants (Nagy et al., 2005).

Two of the protective perturbagens identified in zebrafish, AEG 3482 and AT13387, did not protect hair cells against neomycin-induced death in the mouse adult utricle. It is important to note that in hair cells from both animals, the duration and timing of perturbagen exposure and the dose and duration of exposure to ototoxin must be considered as factors that may cause individual compounds to elicit a protective effect. In previous studies the heat shock stimulus has been administered as a pre-treatment (Cunningham and Brandon, 2006), suggesting that the timing of perturbagen exposure compared to ototoxin application is an additional factor that should be considered. Previous work suggests that zebrafish neuromast hair cells respond to treatment with different aminoglycosides with differential time courses of cell death (Owens et al., 2009), allowing for differentiation between ‘acute’ and ‘chronic’ types of ototoxicity and otoprotective responses (Coffin et al., 2013b). We only tested perturbagens against acute aminoglycoside exposure, and it is possible that they may show a positive response under different conditions. Finally, it may be that perturbagens designed to function against mammalian targets may be ineffective against zebrafish while still effective in utricle cultures.

In summary, we used the transcriptional signature of heat shock, which is protective against ototoxic drug-induced hair cell death, to look for small molecules in the LINCS database that mimic the transcriptional signature of heat shock and thus may also be protective in the inner ear. We heat shocked cultured utricle explants from adult mice and performed RNA-sequencing on them, comparing to control (no heat shock) utricles. We then input selected differentially expressed genes into the LINCS query tool and selected a subset of small molecule perturbagens that either matched, did not match, or reversed the heat shock signature in the cell lines tested in the LINCS database. We screened these molecules for protection against hair cell death caused by the ototoxic aminoglycoside antibiotic neomycin in zebrafish lateral line neuromasts. From this screen, three molecules were protective against neomycin-induced hair cell death: AEG 3482, Pifithrin-μ, and AT13387. The LINCS-identified matching perturbagen pifithrin-μ reproduced the heat shock gene expression signature in cultured mouse utricles, while the LINCS-identified reverse perturbagen AZD-6482 did not induce the heat shock transcriptional signature in utricles. We tested the perturbagens that were protective in the zebrafish screen to determine if they were protective against neomycin-induced hair cell death in cultured utricles. One of the perturbagens, pifithrin-μ, protected hair cells from neomycin damage in the cultured utricle explant model system. Taken together our data describe a new workflow for utilizing RNA-Seq datasets coupled with the LINCS query tool to identify compounds that mimic (or reverse) a gene expression signature of interest for studies of inner ear damage and protection.

## Accession Numbers

RNA-Seq data can be accessed at accession number: GSE116515. Repository name: “The inner ear heat shock transcriptional signature identifies otoprotective compounds against aminoglycoside ototoxicity.”

## Availability of Data

LINCS is an open source, collaborative initiative available at the website (https://clue.io/). For peer-review only: To review GEO accession GSE116515; Go to https://www.ncbi.nlm.nih.gov/geo/query/acc.cgi?acc=GSE116515; Enter token wzwlmsuaxxktlmn into the box. To review aligned files using the UCSC genome browser; Go to http://genome.ucsc.edu/cgi-bin/hgTracks?db=mm10&hgct_customText=https://hpc.nih.gov/GCBCNIDCR/Ryals/all_tracks.txt.

## Author Contributions

MR conceived of the project, performed the experiments, analyzed the data, and wrote the manuscript. RM and DM performed RNA-Seq demultiplexing and alignments and data analysis and critiqued the manuscript. EB performed library preparation and sequencing and critiqued the manuscript. PW performed the experiments and critiqued the manuscript. DR provided guidance and oversight of the zebrafish screening experiments and critiqued the manuscript. LC directed the project, assisted with data analysis, and edited the manuscript.

## Conflict of Interest Statement

The authors declare that the research was conducted in the absence of any commercial or financial relationships that could be construed as a potential conflict of interest.

## Footnotes

1 http://data.lincscloud.org.s3.amazonaws.com/index.html

2 https://clue.io

## Supplementary Material

Figure S1 A Pearson correlation heatmap matrix of gene expression changes in heat shock signature genes in cultured utricles following exposure to either heat shock, AEG3482, Pifithrin-μ, AT13387, AZD-6482, or DMSO vehicle. The color shading within the heatmap corresponds to the degree of correlation. Red indicates a positive correlation coefficient between two treatments; white signifies a lack of correlation, and blue signifies a negative correlation coefficient.

Table S1 The gene names recognized by the LINCS query tool. The 115 enriched genes recognized are displayed in the top half of the table, and the 28 depleted genes recognized are displayed in the bottom half. Genes are displayed in alphabetical order for both enriched and depleted categories.
