# Supplementary figures and images for: The Inner Ear Heat Shock Transcriptional Signature Identifies Compounds That Protect Against Aminoglycoside Ototoxicity
Source: Front Cell Neurosci. 2018 Nov 23;12:445. doi: 10.3389/fncel.2018.00445 (PMC6265442; doi:10.3389/fncel.2018.00445)

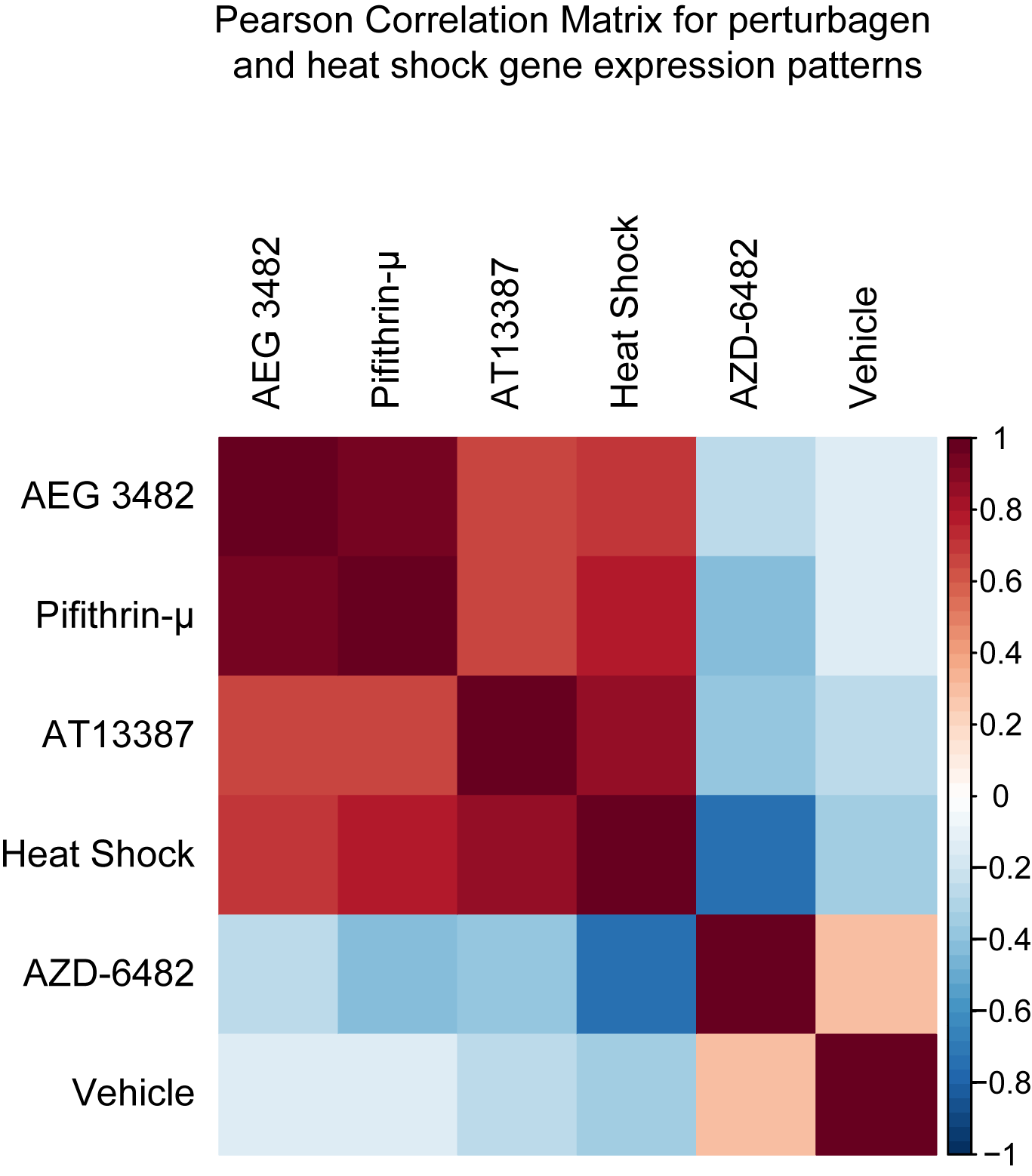

Supplement: Figure S1 — A Pearson correlation heatmap matrix of gene expression changes in heat shock signature genes in cultured utricles following exposure to either heat shock, AEG3482, Pifithrin-μ, AT13387, AZD-6482, or DMSO vehicle. The color shading within the heatmap corresponds to the degree of correlation. Red indicates a positive correlation coefficient between two treatments; white signifies a lack of correlation, and blue signifies a negative correlation coefficient. [file Image_1.TIF]

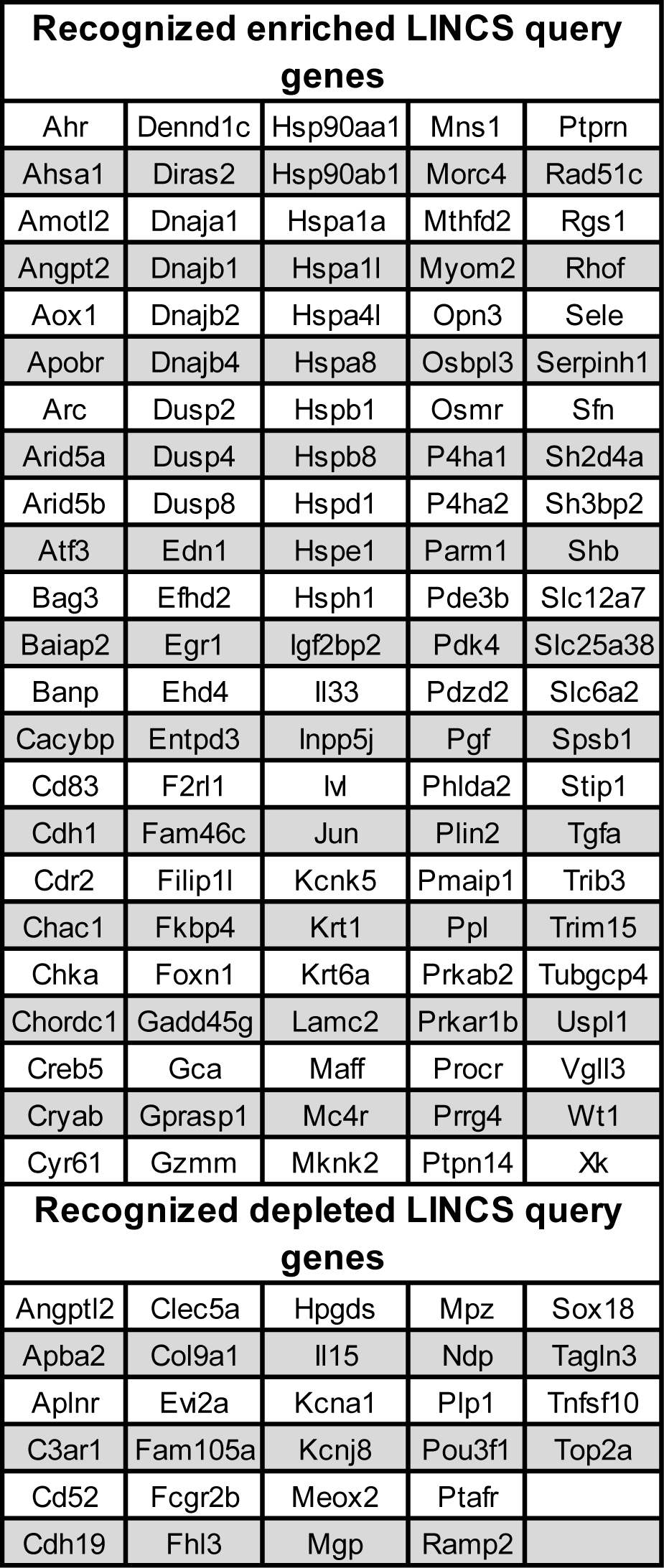

Supplement: Table S1 — The gene names recognized by the LINCS query tool. The 115 enriched genes recognized are displayed in the top half of the table, and the 28 depleted genes recognized are displayed in the bottom half. Genes are displayed in alphabetical order for both enriched and depleted categories. [file Image_2.TIF]
